# Validation of a Lab-on-Chip Assay for Measuring Sorafenib Effectiveness on HCC Cell Proliferation

**DOI:** 10.3390/ijms222313090

**Published:** 2021-12-03

**Authors:** Emanuele Piccinno, Anna Grazia Monteduro, Francesco Dituri, Silvia Rizzato, Gianluigi Giannelli, Giuseppe Maruccio

**Affiliations:** 1 National Institute of Gastroenterology “Saverio de Bellis”, IRCCS Research Hospital, Castellana Grotte, 70013 Bari, Italy; emanuele.piccinno@unisalento.it (E.P.); francesco.dituri@irccsdebellis.it (F.D.); 2 Omnics Research Group, Department of Mathematics and Physics “Ennio De Giorgi”, Institute of Nanotechnology CNR-Nanotec and INFN Sezione di Lecce, University of Salento, Via per Monteroni, 73100 Lecce, Italy; annagrazia.monteduro@unisalento.it (A.G.M.); silvia.rizzato@unisalento.it (S.R.)

**Keywords:** cell proliferation, drug screening, hepatocellular carcinoma, Sorafenib, lab-on-chip, on-chip assays, electrochemical impedance spectroscopy, microfluidics

## Abstract

Hepatocellular carcinoma (HCC) is a highly lethal cancer, and although a few drugs are available for treatment, therapeutic effectiveness is still unsatisfactory. New drugs are urgently needed for hepatocellular carcinoma (HCC) patients. In this context, reliable preclinical assays are of paramount importance to screen the effectiveness of new drugs and, in particular, measure their effects on HCC cell proliferation. However, cell proliferation measurement is a time-consuming and operator-dependent procedure. The aim of this study was to validate an engineered miniaturized on-chip platform for real-time, non-destructive cell proliferation assays and drug screening. The effectiveness of *Sorafenib*, the first-line drug mainly used for patients with advanced HCC, was tested in parallel, comparing the gold standard 96-well-plate assay and our new lab-on-chip platform. Results from the lab-on-chip are consistent in intra-assay replicates and comparable to the output of standard crystal violet proliferation assays for assessing *Sorafenib* effectiveness on HCC cell proliferation. The miniaturized platform presents several advantages in terms of lesser reagents consumption, operator time, and costs, as well as overcoming a number of technical and operator-dependent pitfalls. Moreover, the number of cells required is lower, a relevant issue when primary cell cultures are used. In conclusion, the availability of inexpensive on-chip assays can speed up drug development, especially by using patient-derived samples to take into account disease heterogeneity and patient-specific characteristics.

## 1. Introduction

The development of effective antitumor drugs and personalized therapies is a key focus of modern biomedical and pharmaceutical research. Cancer is the leading cause of mortality in the world after cardiovascular diseases [1]. Liver tumors are the fifth and seventh most frequently diagnosed world cancer in men and women, respectively, and the second and sixth in terms of mortality [2]. Overall, among all primary liver tumors, hepatocellular carcinoma (HCC) is the main histological subtype (80–90%), with an incidence of about 750,000 new cases per year [3,4]. One of the most important factors to define the biological aggressiveness of hepatocellular carcinoma is the tumor growth rate, defined by the doubling time of the neoplastic mass, which varies according to the different degree of cytoproliferative activity and fluctuations over time [5]. In this context, cell proliferation assays are a cornerstone in discriminating physiological from pathological conditions and assessing cancer cell aggressiveness, since an uncontrolled growth rate outside the normal limits is distinctive of cancer cells. Thus, it is essential to discriminate the proliferative kinetics of the cancer cells in order to detect and quantify both the invasiveness potential and the efficacy of drug treatments employed to combat it.

Traditional cell proliferation and drug screening assays based on cell cultures present severe limitations since they are laborious, time-consuming, and expensive as they require continuous work by specialized personnel over several days. Furthermore, they are generally destructive, because measurements performed at the end of the process irreparably alter cellular integrity. Thus, they are not able to monitor continuous changes in the investigated sample and tend also to be operator-dependent.

Here, we report on the optimization and validation of a lab-on-chip for performing cell proliferation and drug screening assays based on electric cell–substrate impedance spectroscopy (ECIS) [6]. This is a non-destructive, label-free approach allowing dynamic high-throughput assays and a continuous monitoring of the investigated biological process to identify the onset of anomalies. ECIS platforms have been shown to be able to monitor motion, attachment, growth, spread, and differentiation of cultured cells [7,8,9], quantifying cell viability and heterogeneity [10,11], as well as cell migration and invasive activities [12,13,14], and evaluating the effects of biochemical compounds and cytotoxicity [15,16,17,18]. Recently, close attention has been devoted to applications for drug research/screening [11,19,20,21,22,23,24,25,26]. In our study, a highly reliable platform for ECIS cell proliferation and drug screening assays is presented, validated by assessing the efficacy of *Sorafenib* for the treatment of HCC. Notably, on-chip results are in agreement with the output of traditional tests performed by crystal violet assays. Moreover, the miniaturized platform presents several advantages in terms of lesser reagents consumption, operator time, costs, and automation. This optimized platform can be employed for a prompt detection of the emergence of chemoresistance and for the investigation of personalized therapies by employing patient-derived samples. The availability of automated inexpensive on-chip assays could revolutionize the field, providing novel possibilities for high-content research and facilitating the emergence of precision medicine and personalized therapies using patient-derived samples to take into account disease heterogeneity and patient-specific characteristics.

## 2. Results and Discussion

### 2.1. Study Design

The scope of this work is to demonstrate a reliable, automated on-chip platform for performing cell proliferation and drug screening assays based on electric cell–substrate impedance spectroscopy (ECIS) and its application for investigating the time-dependent effect of different concentrations of *Sorafenib* on HLF cells. *Sorafenib* is a multi-kinase inhibitor used as a model anticancer drug for the treatment of patients with advanced renal cell carcinoma (RCC) and with unresectable hepatocellular carcinoma [27].

To demonstrate the reliability of our platform for use in targeted assays, we carried out the following phases:

- optimization of chip design, fabrication, materials and platform sterilization;
- validation of ECIS cell proliferation assays with interdigitated electrodes;
- simultaneous monitoring of cell proliferation by optical imaging and impedance spectroscopy to correlate the data;
- control experiments to demonstrate that the trends observed in impedance curves are strictly correlated to cell proliferation;
- dose-dependent on-chip *Sorafenib* drug response assays, validated with crystal violet dissolution cell proliferation assays.

### 2.2. Chip Design and Fabrication

Our lab-on-chip platform for cell proliferation assays and drug screening is based on the combination of a sensing module and a fluidic chamber, modifying a previously reported chip design [16,28,29].

More in detail, the sensing module consists of four arrays, each containing four interdigitated microelectrodes as transducers to perform repeated experiments and statistical analysis. Photolithography and a lift-off process were employed to fabricate the microelectrodes with 10 µm finger/space pitch features, made of thermally deposited Cr/Au (3 nm/30 nm) metal layers on (EOT) glass substrates. The metal thickness was chosen to obtain semi-transparent electrodes allowing optical inspection by means of an inverted microscope.

**Figure 1:**
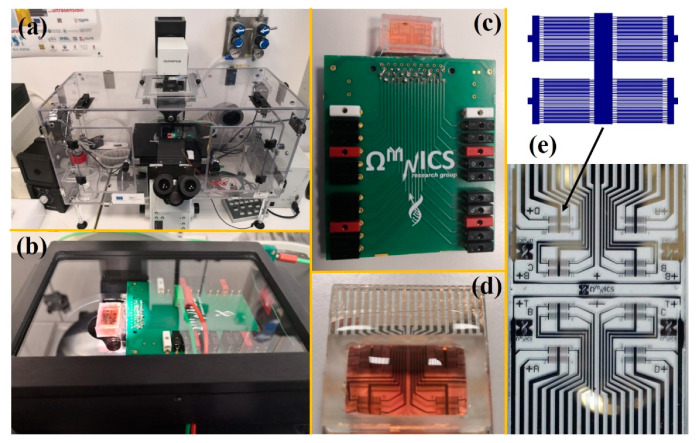
(**a**) Experimental setup employed for on-chip ECIS cell proliferation assays, including a LCR meter for impedance measurements and an inverted optical microscope with a controlled environment chamber for maintaining appropriate values of relative humidity, CO_2_, and temperature. (**b**) Detail of the incubation chamber containing (**c**–**e**) the chip and its interfacing PCB; each interdigitated microelectrode sensor with 10 µm finger/space pitch features covers a 1 × 1 mm area on the chip.

The fluidic chamber was a Petri dish in the initial design but was then replaced with a 3D-printed biocompatible chamber (1 mL volume) sealed on the chip and hosting the cell solution/medium (Figure 1). A crucial step was accurate cleaning of the chip surface to avoid any negative effect on cell proliferation. For this purpose, after fabrication, the chip was first immersed in a 1:1 acetone and isopropanol mixture solution under gentle agitation for 1 min, followed by exposure to nitrogen flow to dry. Then, after assembly, the device was leakage tested with distilled water for 45 min and treated with 75% ethanol and UV rays for 30 min to sterilize the platform and make it ready for the cell seeding.

### 2.3. Optimization and Validation of the On-Chip ECIS Proliferation Assay

As a first step for enabling drug screening studies, the on-chip proliferation assay was optimized and validated. For this purpose, after seeding, cell attachment and spreading on the sensing electrodes were monitored by impedance measurements and optical imaging in order to compare the acquired data and gain further insight and validation. The investigation lasted for a period of about 72 h, which allowed the cell layers to become confluent.

Initially, the AC excitation voltage was optimized to 1 mV, which allowed a good signal-to-noise ratio without affecting the cell growth. Then, a frequency-dependent investigation from few Hz to hundreds of kHz was carried out in order to select the most appropriate frequency to monitor the cell proliferation process. In this study, we found a frequency of around few tens of kHz to be the most sensitive to an increasing number of adherent cells and thus the most suitable for ECIS proliferation assays. Indeed, at high frequencies, the impedance of the cell membrane is relatively small, and the current preferentially flows mainly through the membranes within the insulating cell monolayer. On the other hand, for low frequencies, the membrane impedance is high, and most of the current flows paracellularly through extracellular matrix proteins, through the tight junctions of adherent cells and through the medium (electrolyte solution). As a consequence, all the successive studies were performed while recording the impedance signals at 40 kHz under 1 mV voltage amplitude.

**Figure 2:**
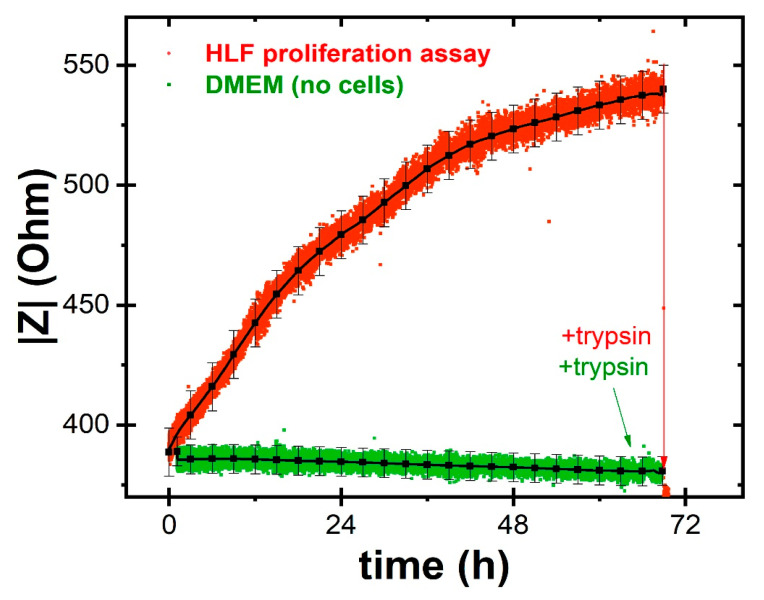
Results of on-chip HLF proliferation assays performed with the ECIS technique. The red curve corresponds to data acquired during HLF proliferation over a three days’ period: the impedance values increase toward a stationary phase and then return to about the initial values once trypsin is added to detach the cells. The green curve is associated to the control experiments with only culture medium and exhibits a practically constant trend. Black curves show mean ± standard deviation including other ECIS sensors in the statistical analyses.

Figure 2 shows the results of an on-chip ECIS assay; the red curve corresponds to HLF cell proliferation data. The recorded impedance modulus was observed to increase from about 400 Ω to about 550 Ω (i.e., about 38% increase), reflecting the cell proliferation behavior. Notably, an increased coverage is expected to result in a larger impedance since the cells act as an insulating layer. In fact, when the cells adhere, spread, and proliferate on the electrodes, the flow of the ionic current is obstructed and the detected impedance value increases. After the initial cell seeding, there is a short lag phase followed by an exponential growth phase in which there is active cell proliferation and, therefore, a substantial increase in impedance. When the cells have formed a semi-confluent monolayer, the exponential increase tends to stop at approximately 45 h post seeding, and a kind of plateau is observed, which corresponds to the stationary phase of the cell cycle. Minor impedance changes associated to cell micromotion can be observed on top of this general trend.

**Figure 3:**
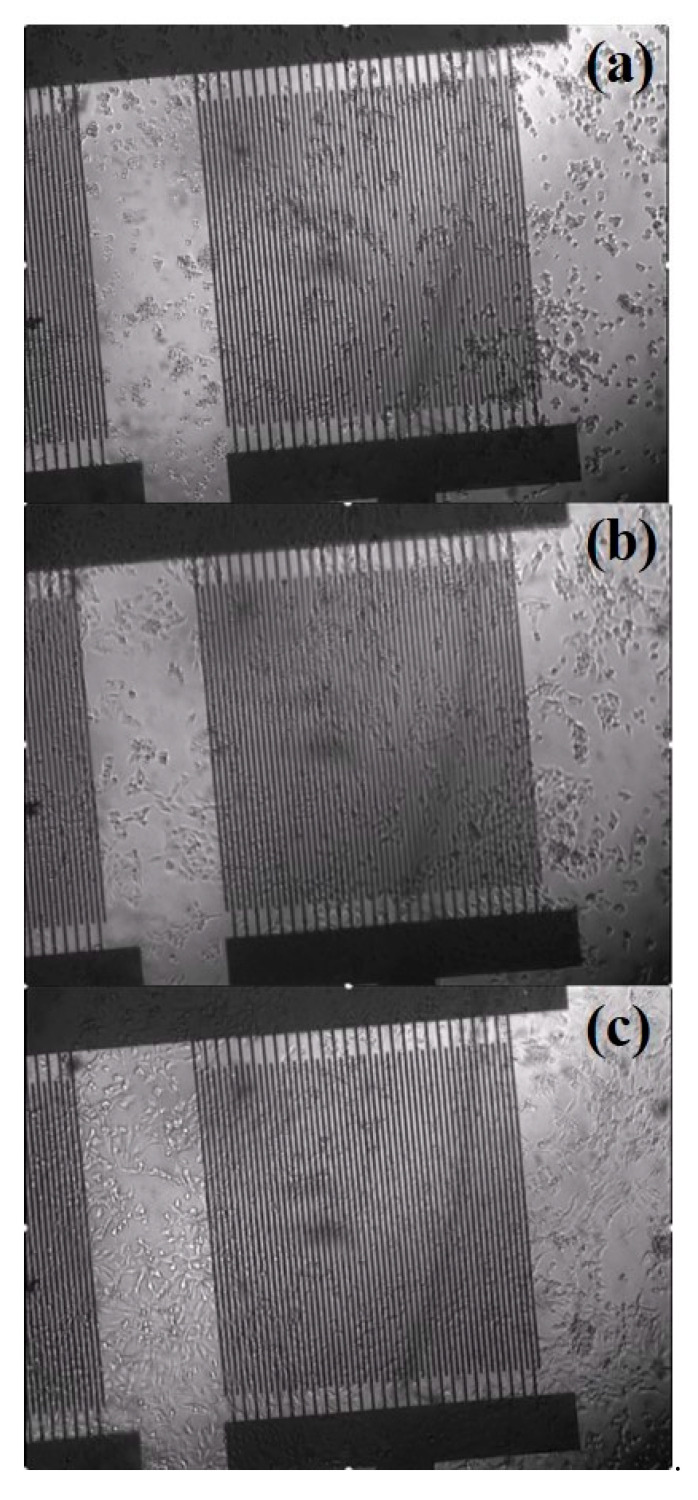
Time-lapse images of cell attachment and spreading on the on-chip platform for ECIS proliferation assays: (**a**) at the beginning of the HLF proliferation assay, immediately after the cell seeding; (**b**) during the exponential growth phase; (**c**) semi-confluent monolayer acquired 45 h post seeding.

These data were confirmed by the images acquired simultaneously with the inverted microscope included in the experimental setup. In Figure 3, three different images are reported, which correspond to the initial phase after cell seeding (Figure 3a), an intermediate moment during exponential growth (Figure 3b) and a period when the cell monolayer was confluent (Figure 3c). Comparing these images in sequence, it is possible to observe how the cells initially start to adhere on the chip and then spread over the substrate, covering a larger and larger area. The decrease in the free area of the electrode causes the measured increase in impedance compared to the value acquired in the absence of cells.

To fully validate our on-chip platform for ECIS cell proliferation assays, we carried out two control experiments. On one hand, we measured the impedance of the culture medium (DMEM), alone or added with DMSO (the *Sorafenib* solvent). The results are shown in the green curve reported in Figure 2. We found that the detected impedance values remained practically stable for the entire duration of the experiment, except for a minor decrease that can be ascribed to some evaporation of the media over time. Furthermore, even the addition of fresh media or other chemical compounds like trypsin did not interfere with the electrical parameters of the initial solution. Thus, we conclude that the trends observed in the red curve can indeed be attributed to cell proliferation.

As a second validation, in some HLF proliferation experiments, we added a concentrated trypsin solution (10×) toward the end of the time-lapse run, once confluence was reached, at about 68 h post-seeding in the red curve reported in Figure 2. It is worth noting here that the detected impedance instantly returns toward the starting values, as a consequence of cellular detachment from the electrodes surface. This last observation clearly demonstrates that the impedance signal is strictly correlated to the proliferation of HLF cells over the electrodes and morphological changes in the monolayer.

### 2.4. On-Chip Assessment of Sorafenib Effectiveness on HCC Proliferation

**Figure 4:**
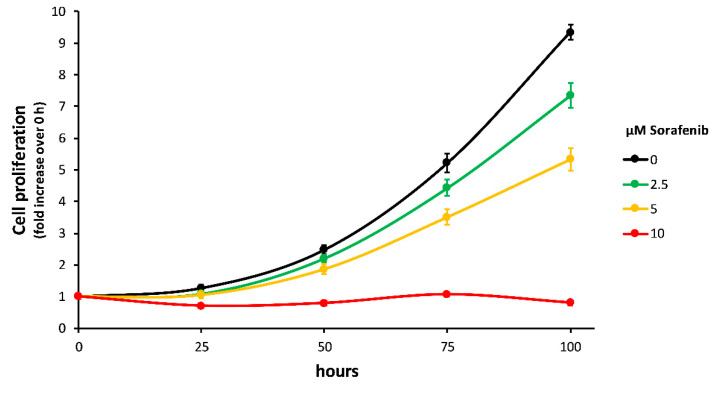
Traditional crystal violet cell proliferation assay. HLF cells were seeded and treated with scaled concentrations of *Sorafenib*. At the indicated time points, the cells were fixed and stained with crystal violet dye. Proliferative index was normalized to time = 0 h.

After the optimization and validation of the on-chip ECIS proliferation assays, we carried out experiments to assess the efficacy of *Sorafenib* as anticancer drug against HLF cells. Specifically, we started from results of traditional crystal violet dissolution cell proliferation assays performed to investigate the effect of *Sorafenib* administration at various concentrations (0, 2.5, 5, and 10 µM in DMSO) on cell growth. Data are shown in Figure 4. The assay was performed as previously reported (Bergamini et al., 2007). Briefly, HLF cells were seeded in triplicate onto wells of a 96-well plate and allowed to adhere overnight. The day after, the medium of wells corresponding to the 0 hours’ time point (baseline value) was removed and cells were fixed with 50 µL of paraformaldehyde (PFA, 4% in PBS) for 15 min, while the medium of cells corresponding to the other time points was replaced with fresh medium (with/without *Sorafenib*). The PFA was then removed, and the cells were stained with crystal violet solution for 10 min. The crystal violet was finally removed, and the wells were abundantly washed with distilled water to remove excess dye, left to dry out, and stored in the dark. This procedure was repeated for cells at the 25, 50, 75, and 100 h’ time points, as well as for empty wells (negative control). At the end of the time course, 100 µL of 1% sodium dodecyl sulphate (SDS) solution was added to the wells to solubilize crystal violet and absorbance was read at 595 nm. Negative control wells were used for background subtraction. Absorbance values were intended as proportional to cell number.

**Figure 5:**
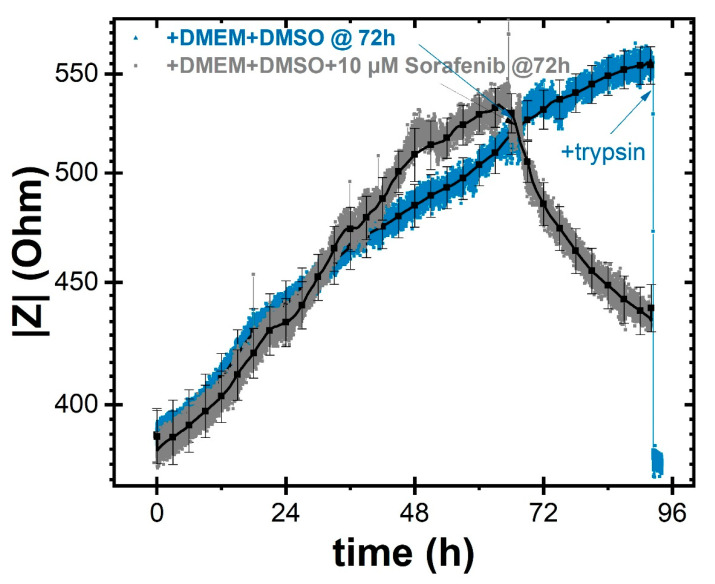
Evaluation of the effect of DMSO solvent (blue curve) and a 10 µM Sorafenib solution (gray curve) on cell proliferation upon administration about three days after cell seeding. Black curves show mean ± standard deviation from statistical analyses.

The first assays performed on the ECIS chip were done to evaluate the effect of *Sorafenib* dissolved in DMSO at a final concentration of 10 µM and administered after about three days from cell seeding. As visible in the gray curve reported in Figure 5, the impedance values started to drop, with a decrease of about 100 Ω per day, as a consequence of altered cell viability. These data demonstrated the effectiveness of the drug, which is able to act on the strength of cell adhesion, morphology, and vitality, causing detachment from the sensing area. *Sorafenib* is, in fact, a drug known to induce cell apoptosis. In this way the increase in the free electrodes area results in a decrease in impedance (on a slower time scale as compared to trypsin administration, blue curve in Figure 5). Remarkably, if after three days we add only the medium with the DMSO solvent (as indicated by the arrow on the blue curve), the effect on the cell proliferation trend appears negligible. This clearly indicates the role of *Sorafenib* in combating cell proliferation.

**Figure 6:**
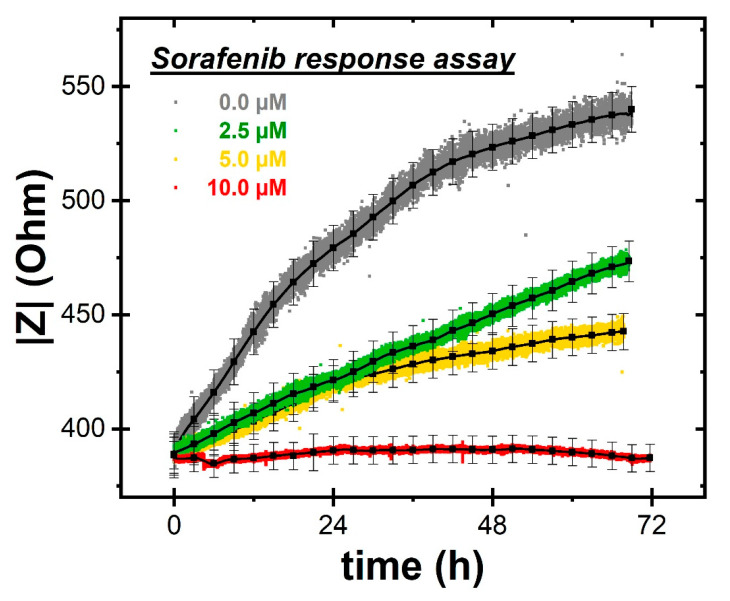
Dose-dependent ECIS cell proliferation assays showing the influence of the *Sorafenib* concentration on the proliferation rate. Gray curve is in absence of *Sorafenib*, while green, yellow and red curves are at increasing *Sorafenib* concentrations from 2.5 µM, to 5.0 µM and 10.0 µM respectively. Black curves show mean ± standard deviation from statistical analyses.

Finally, a dose-dependent study was carried out to investigate the effect of exposing HLF cells to different concentrations of the anticancer drug *Sorafenib*. On-chip ECIS proliferation results (Figure 6) were then compared with traditional assays at the same concentrations. The set of curves exhibits an evident dose-dependent behavior of HLF proliferation, evaluated on the basis of cell impedance increases. Indeed, compared to the reference (gray) curve collected in absence of the drug, the increasing *Sorafenib* concentration clearly led to a declining proliferation trend, which appeared to be almost completely quenched at the 10 µM concentration. Comparing these results with those reported in Figure 4 for traditional crystal violet assays, we can observe a strong analogy. Both methodologies were in agreement that a lethal dose (LD50) of the drug is at about a 5 µM concentration, at which the cell growth rate is almost halved.

Although the results of the two techniques were comparable, the on-chip platform provided important advantages, thanks to the miniaturization and automation of the assay under the control of dedicated software and without the need for continuous monitoring by specialized personnel. In this respect, in Table 1, we report a comparison among estimated reagents consumption, operator time, and assay costs. Notably, on-chip costs can be further reduced by a factor *N*, just by exploiting multiplexing to carry out *N* assays simultaneously (indeed, the reported costs are for the whole chip but in its current design, it could already carry out quadruplicate assays in each one of the four chambers, with suitable fluidics separating them). Furthermore, performing duplicate and comparative assays simultaneously on the same chip can also increase the reliability of the study.

## 3. Materials and Methods

### 3.1. Cell Cultures and Proliferation Assay

All the chemicals necessary for the cell cultures were purchased at the highest degree of purity possible from Sigma (Sigma-Aldrich, St. Louis, MO, USA). The HLF cell line was purchased from JCRB Cell Bank (Japan), and the cells were grown in Dulbecco’s modified Eagle’s medium (DMEM, Sigma-Aldrich, St. Louis, MO, USA), supplemented with 10% FBS (Fetal Bovin Serum, Sigma-Aldrich, St. Louis, MO, USA); 1% of 200 mM L-glutamine (Sigma-Aldrich, St. Louis, MO, USA); 1% antibiotics (10,000 µg/mL streptomycin and 10,000 U/mL penicillin, Sigma-Aldrich, St. Louis, MO, USA), in order to prevent possible bacterial contamination; and 1% of 100 mM sodium-pyruvate (Sigma-Aldrich, St. Louis, MO, USA). The medium was renewed every 2 days, and the cells were replated using a trypsin solution (Sigma-Aldrich, St. Louis, MO, USA) after reaching 80% confluence, and incubated in a humidified incubator, at 37 °C and 5% CO_2_. Standard proliferation assays were performed as previously described [30]. *Sorafenib* was used as previously reported [31].

For on-chip proliferation assays, a suspension of cells (concentration 10^5^ cells/mL) was seeded into the device. For cytotoxicity assays, a DMSO solution containing the anticancer drug *Sorafenib* (BAY-43-9006 Nexavar, Bayer Pharmaceuticals Corp., Leverkusen, Germany and Onyx Pharmaceuticals Inc, Newbury Park, CA, USA) was added, and its effect on the cell proliferation process was monitored. HLF cells were treated with different concentrations of *Sorafenib* (0, 2.5, 5, and 10 µM), and their proliferation and morphological changes were measured by ECIS and microscopy imaging during the anticancer drug treatment.

### 3.2. Electrical Connections and Impedance Measurements

To enhance electrical connections, we designed dedicated pads in the chip layout, with a specific pitch to match a commercial connector integrated on a printed circuit board (PCB). For impedance measurements, this PCB was then employed to interface the platform with an Agilent E4980a LCR meter (Keysight Technologies, Milan, Italy), and a sinusoidal AC signal with 1 mV amplitude was applied between the interdigitated electrodes while recording the impedance as a function of time. Data acquisition was performed by means of dedicated home-made software written in Labview (GM-Multiscan).

After seeding the cells, the whole experiment was carried out within a controlled environment chamber (Okolab s.r.l., Naples, Italy), in which the temperature (37 °C) and the percentage of humidity (95%) and carbon dioxide (5%) are continuously monitored and maintained at optimum levels. An inverted microscope (Olympus IX81, Olympus Italia s.r.l., Segrate, Italy) was employed for simultaneously monitoring cell proliferation by optical imaging through the transparent substrate and semitransparent electrodes. In Figure 1a, the experimental setup employed for on-chip ECIS cell proliferation assays is shown, with details on the incubation chamber (Figure 1b) containing the chip and its interfacing PCB (Figure 1c–e).

### 3.3. Statistical Analysis

All experiments were performed at least in quadruplicate, and ECIS data are reported as single experimental curves (in color) with superimposed black curves showing mean ± standard deviation from statistical analyses, also including other devices. However, device-to-device variations in impedance increase were typically comparable to oscillations observed in single experiments and associated to cells micromotion.

## 4. Conclusions

Cell proliferation assays are the gold standard in drug screening, especially in studies involving the evaluation of the curative potential of newly developed anti-cancer drugs and therapies. In this study, we developed a miniaturized on-chip ECIS platform that proved to be very reliable for automating various tests typically performed in laboratories by specialized personnel. In particular, the reported chip has been demonstrated to allow a dynamic, high-throughput evaluation of HLF cancer cell proliferation and of *Sorafenib* drug efficacy. In general, the optimized platform can enable high-throughput and high-content studies on large drug candidate libraries or on combinations of multiple compounds (drugs, bioactive factors, or media) to assess their putative synergistic effect, thus featuring striking advantages over traditional methodologies.

Miniaturization, assay multiplexing, and automation are indeed of great value when the use of expensive reagents or the investigation of multiple conditions are limiting factors. In this respect, on-chip assays permit long-term monitoring; continuous perfusion; and simultaneous testing of various dosages, mixtures, etc. On the other hand, the availability of the described technology might be not common in all the research laboratories, thereby posing a limitation of the wide application of our findings. Nevertheless, scaling-up of this study could drive the development of industrial manufacturing of the device. Further improvements can be achieved by mimicking relevant in vivo microenvironments on the same chip to better reflect the morpho-molecular conditions of human cancer cells, as well as by evaluating drug administration routes through vascularization and other biological barriers. Finally, we propose the use of our validated platform when choosing the most suitable drug in patients with HCC, taking advantage of the limited number of cells needed that could be freshly isolated from biopsy specimens, thus furthering the design of better personalized medicine strategies.

## Figures and Tables

**Table 1:** Comparison among traditional and on chip assays in terms of estimated reagents consumption, operator time, and assay costs.

		Traditional Assay	On Chip Assay *
**Reagents**		<10^5^ cells >5 mL medium ml reagents	<10^5^ cells <1 mL medium µL reagents
**Operator time**		<1 h settings >2 h control and analysis	<1 h settings <0.5 h control and analysis
**Costs**	**Consumables**	<EUR 5	<EUR 1
	**Personnel**	>EUR 120	<EUR 30

* All chip values must be divided by the assay multiplicity *N* with multiplexing.

## Data Availability

Not applicable.
